# Image quality and radiation dose in planar imaging — Image quality figure of merits from the CDRAD phantom

**DOI:** 10.1002/acm2.12649

**Published:** 2019-06-01

**Authors:** Bente Konst, Harald Weedon‐Fekjær, Magnus Båth

**Affiliations:** ^1^ Department of Radiology Vestfold Hospital Trust Tønsberg Norway; ^2^ Oslo Center for Biostatistics and Epidemiology Research Support Services, Oslo University Hospital Oslo Norway; ^3^ Department of Medical Physics and Biomedical Engineering Sahlgrenska University Hospital Gothenburg Sweden; ^4^ Department of Radiation Physics, Institute of Clinical Sciences Sahlgrenska Academy at University of Gothenburg Gothenburg Sweden

**Keywords:** CD‐curve, contrast detail phantom, figure of merit, IQF, planar imaging, precision

## Abstract

**Purpose:**

A contrast‐detail phantom such as CDRAD is frequently used for quality assurance, optimization of image quality, and several other purposes. However, it is often used without considering the uncertainty of the results. The aim of this study was to assess two figure of merits (FOM) originating from CDRAD regarding the variations of the FOMs by dose utilized to create the x‐ray image. The probability of overlapping (assessing an image acquired at a lower dose as better than an image acquired at a higher dose) was determined.

**Methods:**

The CDRAD phantom located underneath 12, 20, and 26 cm PMMA was imaged 16 times at five dose levels using an x‐ray system with a flat‐panel detector. All images were analyzed by CDRAD Analyser, version 1.1, which calculated the FOM inverse image quality figure (IQF_inv_) and gave contrast detail curves for each image. Inherent properties of the CDRAD phantom were used to derive a new FOM h, which describes the size of the hole with the same diameter and depth that is just visible. Data were analyzed using heteroscedastic regression of mean and variance by dose. To ease interpretation, probabilities for overlaps were calculated assuming normal distribution, with associated bootstrap confidence intervals.

**Results:**

The proportion of total variability in IQF_inv_, explained by the dose (R^2^), was 91%, 85%, and 93% for 12, 20, and 26 cm PMMA. Corresponding results for h were 91%, 89%, and 95%. The overlap probability for different mAs levels was 1% for 0.8 vs 1.2 mAs, 5% for 1.2 vs 1.6 mAs, 10% for 1.6 vs 2.0 mAs, and 10% for 2.0 mAs vs 2.5 mAs for 12 cm PMMA. For 20 cm PMMA, it was 0.5% for 10 vs 16 mAs, 13% for 16 vs 20 mAs, 14% for 20 vs 25 mAs, and 14% for 25 vs 32 mAs. For 26 cm PMMA, the probability varied from 0% to 6% for various mAs levels. Even though the estimated probability for overlap was small, the 95% confidence interval (CI) showed relatively large uncertainties. For 12 cm PMMA, the associated CI for 0.8 vs 1.2 mAs was 0.1–3.2%, and the CI for 1.2 vs 1.6 mAs was 2.1–7.8%.

**Conclusions:**

Inverse image quality figure and h are about equally related to dose level. The FOM h, which describes the size of a hole that should be seen in the image, may be a more intuitive FOM than IQF_inv_. However, considering the probabilities for overlap and their confidence intervals, the FOMs deduced from the CDRAD phantom are not sensitive to dose. Hence, CDRAD may not be an optimal phantom to differentiate between images acquired at different dose levels.

## 1 INTRODUCTION

Methods to evaluate quality of the observed image in radiology have frequently been addressed in previous literature.^1^, ^2^, ^3^ ROC Receiver operating characteristic analyses with clinical images are preferable, but often several practical considerations make the use of clinical images difficult, and receiver operating characteristic analyses are time consuming. Evaluation of images of a contrast detail phantom is more convenient, but has less clinical validity and suffers from poor statistical reliability.^2^

Nevertheless, contrast detail measurements are frequently reported as a subject of routine quality control.^4^, ^5^, ^6^ The CDRAD phantom (Artinis Medical Systems, Elst, the Netherlands) is one commercial option for choosing a contrast detail phantom used to assess image quality, and according to the vendor it can be used within the entire range of diagnostic imaging systems, including fluoroscopy and digital subtraction angiography. It is often used for quality assurance aspects, but the vendor also states that it can be used for optimization purposes.^7^ As such, it has been used for comparison of different detector systems,^8^, ^9^, ^10^, ^11^, ^12^, ^13^, ^14^, ^15^, ^16^ monitors,^17^, ^18^, ^19^ and optimization of acquisition parameters, such as tube voltage.^20^, ^21^

The CDRAD phantom is widely used, but few previous publications report the uncertainty of the figure of merit (FOM) derived from the CDRAD results. Most frequently discussed are the intra‐ and inter‐variability of the observers, but the ability to distinguish between images acquired at different dose levels has not yet been addressed. A stable FOM with small variance is of little use if the FOM does not distinguish between groups of images acquired during different imaging conditions.

The FOM derived from CDRAD is called the inverse image quality figure (IQF_inv_), and is an overall image quality index. It is defined as the inverse sum of the products of each diameter and the associated threshold thickness of the object vaguely seen. IQF_inv_ may be a number that can be hard to interpret for practical purposes. Therefore, a more intuitive FOM for describing the results of a CDRAD study may be valuable. In this study, we suggest a new FOM, h, which describes the size of the hole with the same diameter and depth that is just visible, and compare h to the common FOM IQF_inv_. The main purpose was to evaluate the reliability of the results from semi‐automatic analyses of images of a CDRAD phantom. Based on repeated image acquisitions, the probability of assessing an image acquired at a lower dose as better than an image acquired at a higher dose (the possibility of overlap) was determined. Hence, this is a measure of the FOMs sensitivity to dose level.

## 2 THEORY

The general quality of an image might be determined by the combination of the characteristics of spatial resolution, blurring, contrast sensitivity, noise, and artifacts.^4^ The detectability of the details is limited by the entrance photon fluence, and is further degraded by extraneous noise, contrast‐loss, un‐sharpness, etc. arising in the imaging system.

A method for evaluating the spatial resolution and contrast resolution of an imaging system is determination of the contrast‐detail curve (CD‐curve),^4^ also referred to as threshold contrast detail detectability (TCDD). Quality control test equipment such as CDRAD is a dedicated tool to provide threshold contrast‐detail curves. From the images, the size of the just visible object for each contrast is determined and plotted in a diagram. The decision whether a hole is visible or not, is made by either a human observer or an automatic analysis program. The visibility of high‐contrast objects is said to be limited by the MTF (modulation transfer function) of the imaging system. The right side of a contrast‐detail curve relates to low‐contrast objects, and is said to be noise limited.^4^

### 2.1 CDRAD

**Figure 1:**
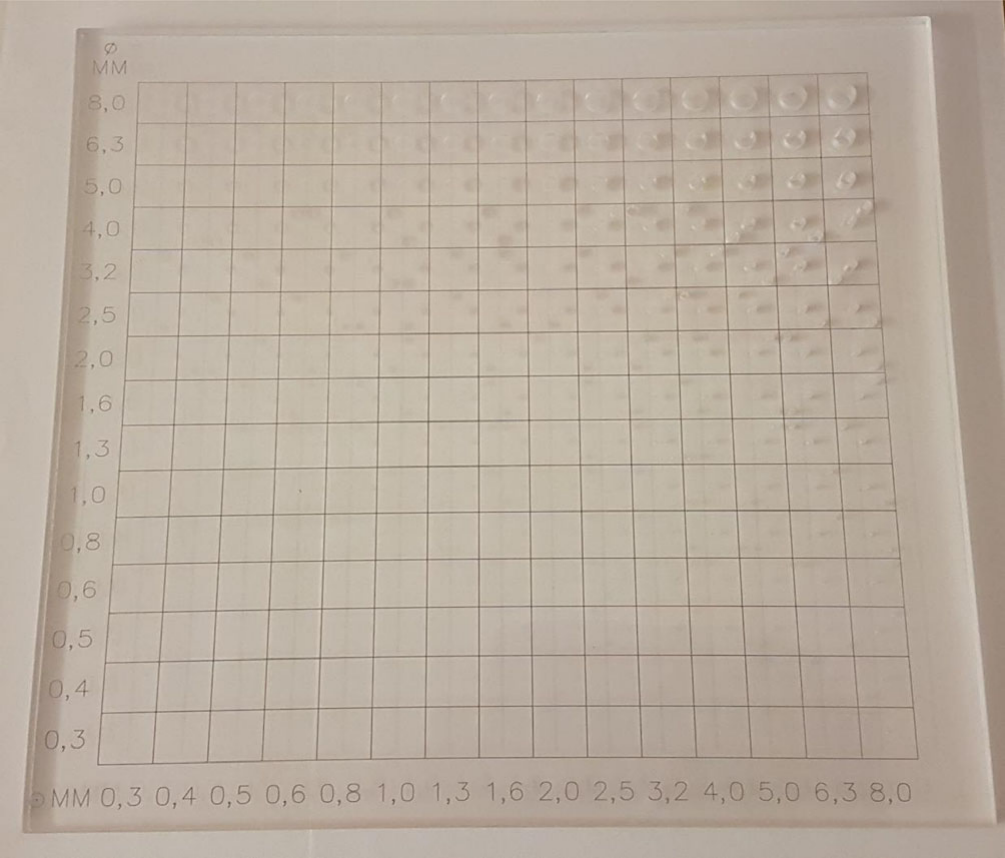
A photo of the CDRAD phantom. The smallest depth and diameter is 0.3 mm, and the largest is 8.0 mm.

**Figure 2:**
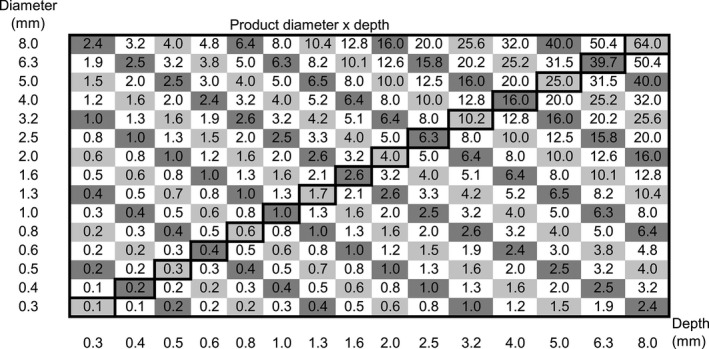
A schematic view of the CDRAD phantom. The x and y axes are the depth (mm) and diameter (mm) of the holes, respectively. The values in the matrix represent the product of diameter and depth. For each diagonal, the product of diameter and depth is approximately equal. The bold boxes represent the main diagonal, where the depth is equal to the diameter.

The CDRAD is designed as an array of 15 × 15 cells with cylindrical holes of different size and depths ranging from 0.3 to 8.0 mm. The depth is constant within each column and the area is constant within a row. Figure 1 shows a photo of the CDRAD. A schematic visualization of CDRAD is given in Fig. 2, which shows that the product of diameter and depth of the holes is almost constant along the diagonals (marked with boxes of the same grayscale). A complete description of the phantom is given in the manual.^7^

X‐ray images of the CDRAD may be automatically analyzed by CDRAD Analyser (Artinis Medical System, Elst, Netherlands). The CDRAD Analyser computes the inverse image quality figure:

(1) $${\text{IQF}}_{\text{inv}}\equiv \frac{100}{\sum_{i=1}^{15}C_{i}\cdot D_{i,th}}$$

where

(2) $$\sum_{i=1}^{15}C_{i}\cdot D_{i,th}=\text{IQF}$$

The image quality figure (IQF) is the sum of the product of depth (*C_i_*) and just visible diameter (*D_i,th_*) across all 15 columns*.* The program computes the average and the standard deviation for both the signal and the background, and uses an unequal variances t‐test to determine if the detail in a certain square is actually seen. The test statistic to decide whether a hole is seen or not, is based on the difference of two means (signal from a hole and signal from the background) and is dependent on a priory difference of means and level of significance.^22^

For calculation purposes, the program applies a rule for a completely not‐scored column (no hole seen for a given depth, regardless of diameter), which results in a *D_i,th_* of 10 mm (the largest phantom diameter is 8 mm). A completely scored column (all hole seen) will result in a *D_i,th_* of 0.3 mm (the smallest phantom diameter is 0.3 mm). The program displays a contrast‐detail curve for each image and a “Group Contrast detail curve” for repeated images (a curve based on interpolation to fit a curve through all images in the group). A reduction in IQF means increased image quality provided that smaller holes and more shallow holes are visible. The inverse IQF represents a FOM where higher value indicates higher image quality. With increased image quality the CD curve will go down.

As mentioned in the introduction part, CDRAD is frequently used for quality control, yet there are few suggested limits. IPEM 2010^6^ recommends the remedial level for a threshold contrast detail detectability to be a deviation of more than 30% of the fitted curve from baseline. Neither the DIMOND III report, “Image quality and dose management for digital radiography”^23^ or the protocol for quality control given by “Quality control of equipment used in digital and interventional radiology”^5^ suggest any limits.

### 2.2 A new FOM h

According to the Rose model, assuming that the contrast is proportional to the depth of a hole, Poisson distributed photons and the noise dominated by quantum noise, the signal‐to‐noise ratio (SNR) is constant when the product of diameter and depth is constant.^24^ Then, the SNR is proportional to the diameter and the depth of the hole as described by Eq. (3).

(3) $$\text{SNR}=\frac{S}{N}\propto \frac{d\cdot A\cdot C}{\sqrt{d\cdot A}}\propto \frac{d\cdot D^{2}\cdot C}{D\cdot \sqrt{d}}\propto D\cdot C\cdot \sqrt{d}$$

where *d* is the dose, *A* is the area, *C* is the depth, and *D* is the diameter of the hole.

As shown in Fig. 2, the product of diameter and depth is approximately constant at the small diagonals of the CDRAD. Given that the assumptions of the Rose model apply, the model implies that if it is possible to observe a given hole on a diagonal of the phantom, it should theoretically be possible to see all holes along this diagonal. Thereby, the CD curve should have a slope of 45° from the upper left to the lower right in Fig. 2 (boxes with the same grayscale).

In this study, a FOM denoted h is defined as the point where the CD curve crosses the main diagonal, as shown with bold boxes in Fig. 2. At this point, the diameter is equal to the depth, and *h* can thus be interpreted as the size of a hole with the same diameter and depth that is just visible. The FOM, *h* is determined from the average *IQF* and defined in Eq. (4):

(4) $$h\equiv \sqrt{\frac{\text{IQF}}{15}}=\sqrt{\frac{100}{15\cdot {\text{IQF}}_{\text{inv}}}}$$

The determination of h is based on the CD curves for each image. To start with, depths where the diameter is set to 10 by CDRAD Analyser, a column without visible holes are excluded 6 and then a new IQFD <10 is determined. Based on this new IQF, h is computed according to Eqs. (5) and (6).

(5) $${\text{IQF}}_{D\;<\;10}=\sum_{\text{Columns}\;<\;10}C_{i}\cdot D_{i,th}$$

(6) $$h=\sqrt{\frac{{\text{IQF}}_{D<\;10}}{\text{number}\;\text{of}\;\text{columns}\;<\;10}}$$

## 3 MATERIALS AND METHODS

A flat‐panel (FP) system from Decotron AS, Norway, was used to produce images of CDRAD. The digital bucky system has a CXDI‐40EG flat‐panel detector (43 cm × 43 cm^2^) from Canon, which consists of a Gd_2_O_2_S scintillation layer mounted on a 2688 × 2688 pixel readout matrix of amorphous silicon. The pixel size is 160 × 160 µm.^25^

The CDRAD Phantom type 2.0 consists of a 26.5 cm × 25.5 cm × 1 cm^3^ plexiglass plate with an array of 15 × 15 cells with cylindrical holes, as described previously.

For investigation of IQF_inv_, the CDRAD 2.0 phantom was exposed lying in contact with the bucky of the FP system, underneath 12, 20, and 26 cm thick PMMA. These PMMA thicknesses simulate a child of about 10 years (30 kg), adult of 60 and 84 kg.

The CDRAD phantom was exposed with a modified chest protocol using 105 kV (child protocol, without grid) and 120 kV (adult protocol with grid) with different mAs and number of replications, according to Table 1. The pixel value is linear with respect to mAs, and all post processing such as edge enhancement, auto adjustment, and noise reduction were turned off. The CDRAD manual recommends at least three repeated images to improve statistics, but a previous publication^26^ recommends seven or more images to keep the intra‐variability of the scores for the individual hole depths below 5%. In addition, Yip et al^27^ recommends 16 images for a similar phantom, CDMAM. Hence, 16 replications at each mAs level were used. In addition, 60 repeated images were acquired at 3.8 mAs for CDRAD with 12 cm PMMA to validate the use of the normal distribution in the statistical computations.

**Table 1:** Exposure settings for CDRAD images. The HVL (half value layer) was measured to 4.7 mm Al for 105 kV and 5.4 mm Al for 120 kV. PMMA gives the thickness of PMMA in addition to the CDRAD phantom. SID is source‐image distance.

PMMA (cm)	SID (cm)	kV	mAs	Field size (cm × cm)
12	183	105	0.8, 1.6 , 1.8^a^, 2.0, 2.5	25 × 25
20	183	120	10, 16, 20^a^, 25 and 32	25 × 25
26	183	120	25, 40, 50^a^, 63 and 80	25 × 25

SID, source‐image distance.

a Is the default mAs chosen by the automatic exposure system.

All images were analyzed using CDRAD Analyser software version 1.1 from Artinis Medical Systems BV. The default settings of CDRAD Analyser were used (a priory difference of means was zero and the alpha level of significance was 1 × 10^−8^), except for the source to bucky distance. The CDRAD Analyser comes up with CD curves and a corresponding *IQF_inv_*_. _Additionally, the new FOM *h* was also determined for each image, according to Eq. 6.

Data from CDRAD Analyser were exported to Microsoft Excel and R (Version 3.3.1, The R Foundation for Statistical Computing, https://www.r-project.org/) for further statistical analyses.

For the data set of 60 repeated images, normal probability Q‐Q plots and 0.05 alpha level Shapiro‐Wilk tests were used to evaluate agreement with the normal distribution. Plots of the FOM vs dose were used to evaluate possible regression models of FOM by dose.

A suitable FOM to evaluate image quality at a given dose has both good separation between dose levels, and low variability within the same dose level. Hence, neither the variation of levels nor the coefficient of variation gives a coherent assessment of the performance of the FOM. The coefficient of determination R^2^, representing the proportion of the total variability of the FOM that is accounted for by the dose, was therefore used to determine which of the FOM is best described by the dose. Correlation analyses showed that the variance was dependent on the dose, and so the condition for linear regression was not fulfilled. Therefore nonlinear regression of both mean and variance was performed. The associated confidence intervals for R^2^ were determined by Jackknife calculations (as many bootstrap samples resulted in nonestimable regression parameters, due to the Bootstrap methods tendency for generating samples with a considerable reduced number of unique observations).

Using the estimated mean and variances from the regression analysis of the FOM, the probability for evaluating an image acquired at a lower dose as better than an image acquired with a higher dose was calculated using numerical integration assuming normal distributions. Repeating the numerical integration for each bootstrap sample, confidence intervals were calculated by percentile bootstrap based on 10 000 bootstrap replications.

## 4 RESULTS

### 4.1 Model observer results

**Figure 3:**
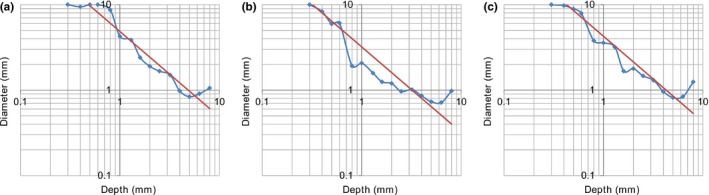
CD curves for a sample of series acquired in this study. The dotted line is the model observer CD‐curve and the straight line is the associated Rose curve, given by h. (a) At 12 cm PMMA, 1.6 mAs, (b) at 20 cm PMMA, 20 mAs and (c) at 26 cm at 50 mAs.

**Figure 4:**
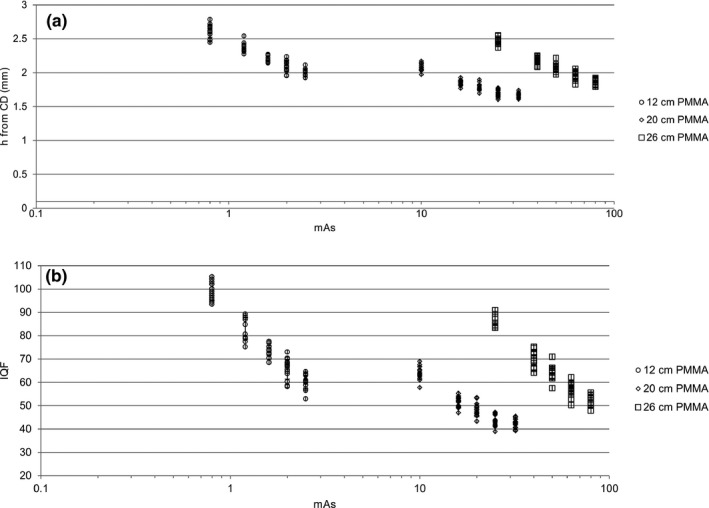
All observed results for: (a) h from CD curves with an error bar representing one standard deviation, based on 16 images and (b) corresponding IQF from CDRAD Analyser.

A few examples of CD curves observed by CDRAD Analyser, and the calculated linear curve based on the Rose model, are shown in Fig. 3. The model observer results deviated from the Rose model for small and deep holes. Figure 4 presents all the observed results for h from CD curves and associated standard deviations and IQF for comparison with previous published findings.

### 4.2 Testing for normal distribution for h

**Figure 5:**
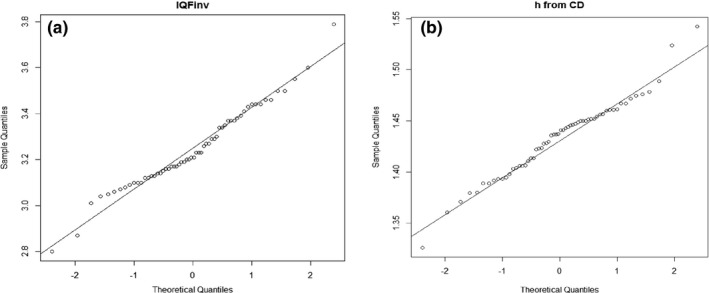
Normal probability plot based on 60 images and 12 cm PMMA: (a) for IQF_inv _og (b) for h from CD curves.

Figure 5 provides Q‐Q‐plots which indicate that the data follow a linear pattern according to the normal theoretical quantiles. All the *P*‐values from the Shapiro‐Wilk tests were greater than 0.05, also within each dose level. It is thus reasonable to assume that both IQF_inv_ and h from CD curves are normally distributed for the setup with 12 cm PMMA. The normal probability plots for h as a function of dose (mAs) (not shown) also provided a linear pattern, but had different mean and standard deviation at each dose level.

### 4.3 Variations in h and IQF_inv_ by dose

The results from normal dispersion regression models for 12, 20, and 26 cm PMMA showed that 8.9%, 11.0%, and 7.8% of the variation in h, and 8.6%, 15.0%, and 9.7% of the variation in *IQF_inv_*, were not explained by dose. Moreover, the regression provided that 8.9%, 11%, and 5.5% of the variance of h was not explained by dose for 12, 20, and 26 cm PMMA. For *IQF_inv_*, the corresponding results were 8.6%, 15%, and 6.6% (Table 2). The significance of difference between R^2^ for h (R^2^(h)) and R^2^ for IQF_inv_ (R^2^(IQF_inv_)) was analyzed. The *P* value and the 95% confidence interval for the difference, R^2^ (h)‐ R^2^( IQF_inv_ )were 0.564 and (0.0354, 0.0289) for 12 cm PMMA, 0.00308 and (0.0125, 0.0716) for 20 cm PMMA and 0.0800 and (−0.00421, 0.0269) for 26 cm PMMA. Hence, R^2 ^(h) was statistically significantly higher for 20 cm PMMA only. But, as can be seen from the confidence interval for 26 cm PMMA, the R^2^(h) was most likely equal or higher than R^2^(IQF_inv_). The small difference between h and IQF_i__nv_ makes it expedient to do further calculation on h only.

**Table 2:** Normal dispersion regression models of *h* as a function of dose (*D*).

Model	Regression result	Model adequacy	95% confidence interval
12 cm PMMA			
*h* = *a + b×D^−1/4^, Var(h)* = *a+bD^−1/2^*	*h* = *0.140 + 2.332* *×D^−1/4^* *Var(h)* = *exp(−6.443 + 1.215×D^−1/2^)*	R^2^ = 0.911 R^2^ = 0.919	(0.880, 0.943)
*IQF_inv_* = *a + b×D^1/2^* *Var(IQF_inv_)* = *a + b×D^−1/2^*	*IQF_inv_* = *0.150 + 0.968 ×D^1/2^* *Var(IQF_inv_)* = *exp(−2.363 – 3.724× D^−1/2^)*	R^2^ = 0.914 R^2^ = 0.964	(0.882, 0.947)
20 cm PMMA			
*h* = *a + b×D^−1/4^, Var(h)* = *a+bD^−1/2^*	*h* = *0.400 + 2.947× D^−1/4^* *Var(h)* = *exp(*−*5.905–0.330×D^‐1/2^)*	R^2^ = 0.890 R^2^ = 0.940	(0.851, 0.930)
*IQF_inv_* = *a + b×D^1/2^* *Var(IQF_inv_)* = *a + b×D^−1/2^*	*IQF_inv_* = *0.458 + 0.358 ×D^1/2^* *Var(IQF_inv_)* = *exp(−1.819 – 11.038× D^−1/2^)*	R^2^ = 0.850 R^2^ = 0.920	(0.791, 0.908)
26 cm PMMA			
*h* = *a + b×D^−1/4^, Var(h)* = *a+b×D^‐1/2^*	*h *= *0.046 + 5.403× D^−1/4^* *Var(h)* = *exp(−5.826 – 0.966×D^−1/2^)*	R^2^ = 0.945 R^2^ = 0.992	(0.922, 0.967)
*IQF_inv_* = *a + b×D^1/2^* *Var(IQF_inv_)* = *a + b×D^−1/2^*	*IQF_inv_* = *0.146+ 0.201×D^1/2^* *Var(IQF_inv_)* = *exp(−1.986 – 23.811× D^−1/2^)*	R^2^ = 0.934 R^2^ = 0.981	(0.903, 0.964)

*a* and *b* are regression coefficients. R^2^ is the coefficient of determination, representing the proportion of the total variability in the outcome that is explained by the dose.

The respective 95% confidence intervals for R^2^ are given.

### 4.4 Assessing images by means of h as a function of dose (mAs)

For 12 cm PMMA the probability for overlap was 1%, 5%, 10%, and 10% for comparing images acquired with 0.8 and 1.2 mAs, 1.2 and 1.6 mAs, 1.6 and 2.0 mAs, and 2.0 and 2.5 mAs, respectively.

For 20 cm PMMA the probability for overlap was 0.5%, 13%, 14%, and 14% for comparing images acquired with 10 and 16 mAs, 16 and 20 mAs, 20 and 25 mAs, and 25 and 32 mAs, respectively.

For 26 cm PMMA the probability for overlap was 0%, 5%, 6%, and 6% for comparing images acquired with 25 and 40 mAs, 40 and 50 mAs, 50 and 63 mAs, and 63 and 80 mAs, respectively.

The probabilities for overlap among the non‐neighbour doses were low or very low (Tables 3, 4, 5). Even though the probability for overlap was small, the 95% confidence interval could be relatively large, see Tables 3, 4, 5 for associated confidence intervals.

**Table 3:** The probability (%) of assessing an image acquired at a lower dose as better than an image acquired at a higher dose for 12 cm PMMA.

mAs	0.8	1.2	1.6	2.0
1.2	1.2% (0.1–3.2) [1.50]		5.1% (2.1–7.8) [1.33]	0.20% (0.0–0.6) [1.67]
1.6	0.00% (0.0–0.1) [2.00]	5.1% (2.1–7.8) [1.33]		10.3% (6.1–13.7) [1.25]
2.0	0.00% (0.0–0.0) [2.50]	0.20% (0.0–0.6) [1.67]	10.3% (6.1–13.7) [1.25]	
2.5	0.00% (0.0–0.0) [3.13]	0.00% (0.0–0.0) [2.08]	0.60% (0.1–1.6) [1.56]	10.5% (5.7–14.6) [1.25]

The respective 95% confidence intervals are given in parenthesis, and the dose factors (DF = mAs_2_/mAs_1_) in brackets.

**Table 4:** The probability (%) of assessing an image acquired at a lower dose as better than an image acquired at a higher dose for 20 cm PMMA.

mAs	10	16	20	25
16	0.46% (0.0*−*3.0) [1.60]		13.0% (8.2*−*17.0) [1.25]	1.4% (0.4*−*2.9) [1.56]
20	0.00% (0.0*−*0.3) [2.00]	13.0% (8.2*−*17.0) [1.25]		14.4% (10.0*−*17.7) [1.25]
25	0.00% (0.0*−*0.0) [2.50]	1.4% (0.4*−*2.9) [1.56]	14.4% (10.0*−*17.7) [1.25]	
32	0.00% (0.0*−*0.0) [3.2]	0.05% (0.0*−*0.2) [2.00]	1.5% (0.4*−*3.1) [1.60]	13.5% (8.4*−*17.6) [1.28]

The respective 95% confidence intervals are given in parenthesis, and the dose factors (DF = mAs_2_/mAs_1_) in brackets.

**Table 5:** The probability (%) of assessing an image acquired at a lower dose as better than an image acquired at a higher dose for 26 cm PMMA.

mAs	25	40	50	63
40	0.00% (0.0*−*0.1) [1.60]		5.1% (2.2*−*7.9) [1.25]	0.1% (0.0*−*0.3) [1.58]
50	0.00% (0.0*−*0.0) [2.00]	5.1% (2.2*−*7.9) [1.25]		5.7% (2.6*−*8.7) [1.26]
63	0.00% (0.0*−*0.0) [2.52]	0.1% (0.0*−*0.3) [1.58]	5.7% (2.6*−*8.7) [1.26]	
80	0.00 % (0.0*−*0.0) [3.2]	0.00% (0.0*−*0.0) [2.00]	0.1% (0.0*−*0.5) [1.60]	6.3% (2.7*−*10.5) [1.27]

The respective 95% confidence intervals are given in parenthesis, and the dose factors (DF = mAs_2_/mAs_1_) in brackets.

## 5 DISCUSSION

In this study, a new FOM originated from CDRAD is outlined, h (the size of the hole with the same diameter and depth that can be discerned on the image), and its variation by dose is compared to the original FOM IQF_inv. _The probability of assessing an image acquired at a lower dose as better than an image acquired at a higher was determined by creating 16 images at five different dose levels for 12, 20, and 26 cm PMMA simulating a child of 30 kg and adults of 60 and 84 kg. Statistical analyses were performed to determine the FOM that best reflects the applied dose, and the associated precision was determined. An optimal FOM is able to differentiate images acquired with quite small dose differences. It is not enough to have a FOM with little variance, as it also must change enough according to the dose.

In conformity with R^2^, the model for h gave a slightly better FOM than IQF_inv_ to predict changes according to the dose. The columns scoring 10 were removed to avoid underestimating the h value. If there are no visible holes in a column, then a 10 mm disc may not be the true threshold. Even though the difference between h and IQF_inv _was minor, and only significant for 20 cm PMMA, h may be a more intuitive parameter than IQF_inv_.

In the experiments, small and deep holes were not seen as expected by the Rose model. The reason for this is probably dose cutoff at x‐ray central beam angles greater than 3°. Ideal x‐ray beams hit the bottom of the hole and go all the way through the cylindrical hole, to the top. But at a small angle from the central beam, the photons that go through the bottom of the hole go sideways through the cylinder wall instead. At even larger angles, none of the x rays will go all the way through the cylinder. The photons that leave the hole through a sidewall will increase the signal in the background and further reduce the contrast. At a few degrees from the central ray the effective contrast may be lower for small deep holes than for shallow small holes. The holes in the CDRAD are parallel like the lamellas in the early design of grids used in planar x‐ray imaging. They were parallel and only available for low grid ratios, for example, grid ratio 6, to avoid the dose cutoff.^28^ To study the influence of centration of the central x‐ray beam, a few images were acquired with the x‐ray central beam on the small deep holes instead of on the central part of phantom. Then, CDRAD Analyser detected the deep small holes. To achieve a better CDRAD phantom, the holes with high ratio should be drilled in the central part of the phantom.

**Figure 6:**
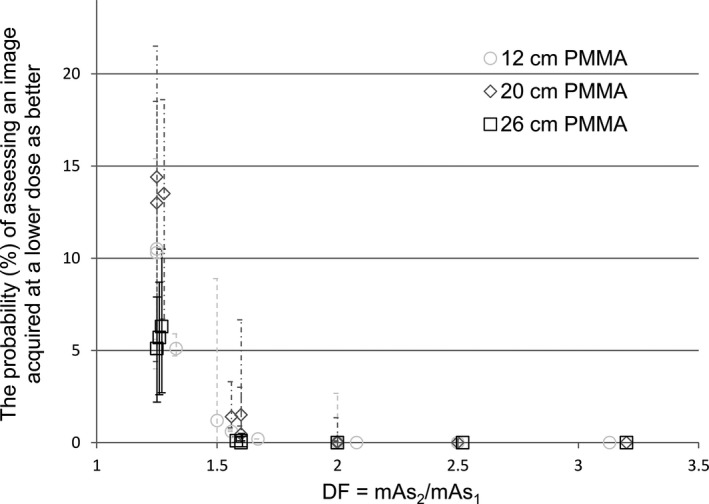
The probability of assessing an image acquired at lower dose as better than an image acquired at higher dose versus the ratio of doses, and the associated confidence interval.

It is expected that the standard deviation of h increases as the value of h increases at lower doses. The measurements showed a nonlinear relation between SD and h, see Fig. 6. This might be due to the fact that the size of the holes increases in a discrete way in the CDRAD phantom, and not continuously. If the dose level corresponds to visualize a hole in between two sizes in the phantom, the standard deviation will suddenly increase. The results in the present study are in good agreement with the results presented in Table 2 in the paper by Alsleem et al.,^29^ performed on CDRAD with 10 cm PMMA. They found a nonlinear relation between SD and dose, and a strong correlation between variance and dose. However, the correlation sign varied between the series with different kV. They also did not find any significant difference in the images of the CDRAD phantom, even with a 100% increase in the dose.

When CDRAD images are analyzed by a human observer, the primary sources of error are relevant: (a) within‐observer variance, (b) between‐observer variance, and (c) sample variance due to different representation of the quantum noise and variations in the imaging processing conditions.^30^ In this study, the images were read automatically by the use of CDRAD Analyser to avoid both intra‐ and inter‐observer variability.^26^, ^31^ It is not known how the CD curves would have been with human observers, but Pascoal et al showed that the IQF_inv_ is reasonably equal, although the curves are different. Pascoal^26^ reported that the slope of the software curves decreased gradually and tended to become approximately parallel to the x‐axis, while the curves of the average observer did not exhibit this feature and showed a straight‐line fashion. Post processing is an important benefit of digital radiography, but it is not suitable to use standard chest post processing on CDRAD images since the processing is dependent on the density of the object. CDRAD has obviously a different content than a chest.^32^ In the present study, all user available post processing was turned off and it was verified that the pixel values in the images were linear to the applied mAs. The noise was somewhat correlated (the noise power spectrum (NPS) was not a straight line) and the shape of the NPS showed some dependency on dose, as expected for a detector based on indirect conversion, but the variations were small enough for the Rose model to be applied with decent validity.

The probabilities for overlapping assessments of the images were quite high (Tables 3, 4, 5). This is in compliance with Loos et al.^33^ regarding CDMAM, a similar phantom for mammography with gold discs instead of holes. Using CDMAM, a change in detection rate (sensitivity) could hardly be observed even when the dose was increased by a factor two (DF = mAs_2_/mAs_1_ = 2). A study using CDRAD and IQF as FOM showed that IQF differences of 10 were significant, and probably true.^12^ In this study, a difference in IQF of 10 was seen between 0.8 and 1.2 mAs (DF = 1.5), and 1.2 and 1.6 mAs (DF = 1.33) for 12 cm PMMA, and between 10 and 16 mAs (DF 1.6) for 20 cm PMMA, and between 25 and 40 mAs (DF 1.6) for 26 cm PMMA. According to difference in IQF it was not possible to differentiate images with DF 1.25 for any of the PMMA thicknesses. There was limitation in the x‐ray tube regarding mAs increase step, therefore it was not possible to acquire images with smaller DF steps. From Fig. 6, it seems that it is easier to differentiate images using 26 cm PMMA than 20 cm PMMA for DF <1.5, and the 12 cm is intermediate. The 20 and 26 cm data are acquired without any movement of the CDRAD, while the 12 cm data was acquired after reposition. The experimental setup may slightly vary, due to uncertainties in the light field. A few images were acquired to find an explanation why deep small holes are more difficult to detect than shallower holes. These images indicate that the CDRADs position with respect to the x‐ray tube may be of considerable importance. But the sensitivity to relative position of CDRAD and x‐ray tube is not addressed in this study.

Using CDRAD it might be difficult to differentiate the images at dose levels where few or no holes are seen even if there is a large increase in the dose. Equally at high doses, where all the holes are seen or a limit in the system is reached, it is difficult to tell one image from the other due to the dose. It might also be difficult when the detector reaches a saturation level. Thus, it is difficult to establish which dose difference is possible to detect using CDRAD, because the noticeable difference depends also on where on the dose scale the images are acquired. Other limitations using CDRAD may be the lack of conformity with the radiologist opinion of patient images,^34^ and the lack of anatomical noise.^15^

## 6 CONCLUSION

The CDRAD phantom and two associated FOMs were evaluated. The results indicate that both *IQF_inv_* and *h* are about equally determined by the dose, but h may be a more intuitive parameter to understand: the smallest hole with equal depth and diameter that is possible to see in the image. The required dose increase to get images for which the probability to assess an image acquired at the lower dose as better is less than 5%, is a 50% at least. Therefore, it is not expected to reliably detect dose variations smaller than 50% using single CDRAD images.

## CONFLICT OF INTEREST

The authors declare no conflict of interest associated with this manuscript.
